# Newborn care practices and its determinants among postnatal mothers in Dessie Referral Hospital, Northeast Ethiopia

**DOI:** 10.1186/s13104-019-4133-3

**Published:** 2019-02-21

**Authors:** Yaregal Semanew, Meaza Etaye, Alemayehu Tizazu, Desalegn Abebaw, Tsegaye Gebremedhin

**Affiliations:** 1 Department of Pediatrics and Child Health Nursing, Wollo University, Dessie, Ethiopia; 2 Department of Health Systems and Policy, University of Gondar, P.O. Box: 196, Gondar, Ethiopia

**Keywords:** Practice, Newborn care, Postnatal mothers, Ethiopia

## Abstract

**Objective:**

Newborn care practices like the initiation of breastfeeding within an hour, delay baby bathing, cord cutting with a safe instrument, and thermal care is a crucial intervention for the avoidance of more than 75% of neonatal deaths. Therefore, this study aimed to describe the practices and determinants of newborn care among postnatal mothers attending postnatal clinics in Dessie Referral Hospital, Northeast Ethiopia.

**Results:**

A total of 418 postnatal mothers included in the study. The finding indicated that the proportion of initiation of breastfeeding within an hour, baby bathing after 24 h, cord cutting with a safe instrument, and thermal care was 83.9%, 75.8%, 46.9%, and 80.8% respectively. Overall, 46.9% (95% CI 41.9, 51.9) of newborn care practices was good. In the multivariable logistic regression analysis; women earning 651–1400 Ethiopian birr monthly (AOR = 0.428; 95% CI 0.183, 0.999), women who delivered spontaneous vaginally for their recent delivery (AOR = 0.438; 95% CI 0.240, 0.800), and mothers who had antenatal follow up once (AOR = 0.111; 95% CI 0.013, 0.944) were factors significantly associated with newborn care practices. Therefore, enhancing antenatal care services and providing counseling for these spontaneously delivered mothers will increase newborn care practices.

**Electronic supplementary material:**

The online version of this article (10.1186/s13104-019-4133-3) contains supplementary material, which is available to authorized users.

## Introduction

Many countries in Sub-Saharan Africa are reported to have made little or no progress towards the child survival target, and some countries had even witnessed a deterioration in child survival rates [1]. Fifty-two percent of neonatal deaths are due to lack of appropriate care given to neonates, and most of those deaths could be prevented through low-cost interventions like quality care at birth and essential newborn care practices [2]. To save the life of neonates, the recommended WHO essential newborn care practices are crucial interventions; which includes clean cord care, thermal protection, early and exclusive breastfeeding, delay bathing, care for the low birth weight newborn and management of newborns [3].

In Ethiopia, suboptimal newborn care practices still persist and neonatal mortality rates have been resistant to change and contributes about 43% of all under-five deaths [4, 5].

The first 28 days of life is the most vulnerable time for a child’s survival with an average global rate of 18 deaths per 1000 live births (LBs) in 2017 [6]. More than 90% of neonatal deaths occur in sub-Saharan Africa (27 deaths per 1000 LBs in 2017) and about half of the deaths occur at home [7].

Ethiopia reported having made little or no progress towards the child survival target which is majorly contributed by neonatal mortality. The neonatal mortality rate (NMR) and postneonatal mortality rate were 29 and 19 deaths per 1000 LBs respectively with the proportions of early 79% and late neonatal deaths 21% [8].

The lack of progress in the reduction of NMR may be explained by the high proportion of birth taking place at home, low availability and poor newborn care practices [9–11]. There are also numerous unscientific and unhygienic health practices and social taboos in neonatal care that makes the newborn extremely vulnerable [12].

The first 48 h after delivery is the time when the mother and baby are most vulnerable to morbidity and mortality associated with childbirth [13, 14].

It is estimated that 75% of neonatal deaths could be avoided with simple, low-cost tools that already exist such as sterile blades to cut the umbilical cord, using clean dry clothes to wrap and keep babies warm, and early initiation of breastfeeding.

A study conducted in Mandura District Northwest Ethiopia shows; 48.1% of mothers began breastfeeding within the first hour of birth and 37.8% bathed their baby after 24 h of birth [9]. A study conducted in four regions of Ethiopia shows bathing during the first 24 h of life (74.7%), application of butter and other substances to the cord (19.9%), and discarding of colostrum milk (44.5%) [10].

Even though there are some studies assessing newborn care practices in Ethiopia, there was no study done in Dessie Referral Hospital, which is the only referral public Hospital that serves for many populations in Northeast Ethiopia. The main aim was to describe the practices and determinants of newborn care among postnatal mothers attending postnatal clinics of Dessie Referral Hospital.

## Main text

### Methods

#### Study design and setting

A facility-based cross-sectional study was conducted to describe the newborn care practices and determinants among postnatal mothers attending postnatal clinics in Dessie referral Hospital from April 13 to May 18, 2018. Dessie Referral Hospital is found in Dessie town, South Wollo Zone 401 km to the Northeast of Addis Ababa, Ethiopia.

The study populations were all selected postnatal mothers who came for postnatal services in postnatal clinics of Dessie referral Hospital during the data collection period. Postnatal mothers with an alive newborn within 42 days after delivery were included, whereas postnatal mothers who were unconscious, seriously ill and mentally retarded were excluded.

#### Sample size and sampling procedures

The sample size was determined using Epi-Info version 7.1 statistical software; using a proportion of mothers having good newborn care practice 50% to get the maximum sample size with a 5% margin of error. Finally, adding a 10% non-response rate the final sample was 423. Systematic random sampling was used with the sampling fraction K^th^ was calculated (k = N/n = 865/423 = 2) (973 mothers have visited the hospital for postnatal service in April 2017 from health management information system report) and the first case was selected by lottery method. Newborn care practice is the dependent variable, whereas sociodemographic and economic factors, parity, history of ANC, place of delivery, mode of delivery, and knowledge of mothers about newborn care were the independent variables. An interviewer-administered structured questioner was first prepared in English, and then translated into the local language (Amharic), and then back to English to maintain consistency. A pretest was conducted on 5% of the sample at Boru-Meda Hospital and then the necessary correction was made. Training was given to both the data collectors and supervisors, and supervisors monitored the data collection process on a daily basis.

#### Measurements

Initiation of breastfeeding: the recommended practice of breastfeeding a newborn baby initiated breastfeeding within 1 h after birth.

Baby bathing: the practice of newborn baby bathing only after 24 h of birth.

Cord cutting: the practice of cutting a cord with a new blade or a boiled blade.

Thermal care: when the newborn was dried and wrapped after birth.

Finally, newborn care practice is dichotomized based on the four newborn care practices mentioned above. Those mothers provided three or more practices were categorized as “good newborn care practices” otherwise they categorized as “poor newborn care practices” [9, 10].

#### Data management and analysis

Data were entered to Epi-Data Version 3.1 and exported to SPSS version 22 for analysis. Factors were tested using the bivariable analysis, and p-value ≤ 0.2 was a candidate for the multivariable logistics regression analysis. Adjusted odds ratio (AOR) with a 95% CI and p-value < 0.05 was used in multivariable logistic regression analysis to show the association between explanatory and dependent variables.

### Results

#### Sociodemographic and economic characteristics of study participants

A total of 418 postnatal mothers responded to the interviewer-administered questionnaire with 98.8% response rate. Of these, 361 (86.4%) were between the age of 21 and 35 years and 236 (56.5%) were multiparous. Moreover, 294 (70.3%) of them were a housewife and 387 (92.6%) were married. Religiously, 255 (61.0%) were Muslim and 90.0% of the respondents had a monthly income of more than 1401 Ethiopian Birr (Table 1).

**Table 1:** Sociodemographic and economic characteristics of postnatal mothers at Dessie Referral Hospital, April 2018 (n = 418)

Variables	Category	Frequency	Percent (%)
Age in years	15–20	13	3.1
	21–35	361	86.4
	36–49	44	10.5
Marital status	Single	11	2.6
	Married	387	92.6
	Divorced	12	2.9
	Widowed	8	1.9
Religion	Orthodox	133	31.8
	Muslim	255	61.0
	Protestant	17	4.1
	Catholic	13	3.1
Ethnicity	Amhara	402	96.2
	Oromo	16	3.8
Educational status	Unable to read and write	57	13.6
	Able to read and write	37	8.9
	Primary education	172	41.1
	Secondary education	91	21.8
	College and above	61	14.6
Occupation	Housewife	294	70.3
	Private employee	28	6.7
	Government employee	42	10.0
	Merchant	42	10.0
	Student	12	3.0
Average monthly income of family (ETB)	151–650 ETB	11	2.6
	651–1400 ETB	31	7.4
	> 1401 ETB	376	90.0

*ETB* Ethiopian Birr

#### Antenatal care and delivery history of study participants

Majority 407 (97.4%) of respondents had ANC follow up at least once. Among these, 295 (72.4%) were started ANC follow up before their 4th months of gestation and 241 (59.2%) of them had visited ANC four times and above. Whereas, 398 (95.2%) have received TT vaccination. Of the total respondents, 411 (98.3%) of postnatal mothers delivered at a health facility and attended by a skilled healthcare provider; whereas, the rest delivered at home. Regarding their mode of delivery; 57.1%, 24.9%, and 18.0% of mothers delivered spontaneously, by cesarean section and instrumentally respectively.

Two hundred thirteen (52.3%) of postnatal mothers did not get information about newborn care during their ANC visits and the remaining; 141 (34.6%), 47 (11.6%) and 6 (1.5%) postnatal mothers got information about breastfeeding, immunization, and thermoregulation respectively. Of these, for 161 (83.0%) mothers the information was provided by a nurse and for 33 (17.0%) mothers by a doctor (Additional file 1: Table S1).

#### Postnatal mothers’ newborn care practices

Majority 286 (68.4%) of mothers placed their baby on the abdomen before the placenta was delivered and 196 (46.9%) of mothers used a new blade to cut the cord after delivery. After the cord was cut, 234 (56.0%) of mothers cover with cloth and 110 (26.3%) of mothers uncover and keep dry. Additionally, 10 (2.4%) of mothers apply anything (butter and vaseline) on the stump after the baby’s’ cord was cut. Moreover, 338 (80.8%) of mothers wrapped the baby with a new cloth immediately to keep their baby warm and 406 (97.1%) of mothers initiate breastfeeding immediately after delivery. Of these, 141 (33.7%) of mothers initiated before 30 min and 210 (50.2%) between 30 min and an hour.

Furthermore, 364 (87.1%) of postnatal mothers cleaned their breast and hands before breastfeeding their baby and 257 (61.5%) of mothers fed their baby 8–12 times per day. Additionally, 317 (75.8%) of mothers bath their baby after 1 day of their delivery and only 97 (23.2%) of mothers immunized their baby at birth. Among these home deliveries majority (71.4%) of mothers practicing newborn care poorly/sub-optimally. But, from those facility born babies, only 47.3% received newborn care optimally. Overall, 222 (53.1% CI 48.1, 58.1) of postnatal mothers’ newborn care practice was poor (Table 2).

**Table 2:** Newborn care practice of postnatal mothers at Dessie referral Hospital, Northeast Ethiopia, 2018

Variables	Category	Frequency	Percent (%)
Where was the baby placed before the placenta was delivered?	On the mother’s abdomen	286	68.4
	On clean surface	128	30.6
	Other	4	1.0
What instrument was used to cut the cord after delivery?	New or boiled blade	196	46.9
	Old and un boiled blade	6	1.4
	Don’t know	214	51.2
	Other^a^	2	0.5
What did you do to the umbilical stump after the cord is cut? (n = 418)	Cover with cloth	234	56.0
	Uncover, keep dry and clean	110	26.3
	I do not know	74	17.7
Did anybody apply anything on the stump after the baby’s cord was cut?	Yes	10	2.4
	No	408	97.6
If yes, what was applied? (n = 10)	Butter	2	20.0
	Vaseline	8	80.0
What did you do to keep your baby warm? (n = 418)	Skin to skin contact	75	18.0
	Wrapped the baby in a cloth immediately	338	80.8
	Both	5	1.2
What was the first feed you gave to the baby immediately after delivery?	Breast milk	406	97.1
	Cow milk	4	1.0
	Formula feed	2	0.5
	Other^b^	6	1.4
When did you start breastfeeding after delivery?)	Immediately	141	33.7
	30 min to 1 h	210	50.3
	Other	67	16.0
Do you clean your breast and hands before breastfeeding the baby? (n = 418)	Yes	364	87.1
	No	54	12.9
How often did you breastfeed your baby in a day?	8–12 times	257	61.5
	On-demand	158	37.8
	Don’t breastfeed	3	0.7
When did you start bathing your baby after birth?	Immediately after birth	9	2.1
	After 6 h of birth	24	5.8
	After 1 day of birth	317	75.8
	Other^c^	68	16.3
Was your baby immunized at birth?	Yes	97	23.2
	No	321	76.8
Overall newborn care practice	Good	196	46.9
	Poor	222	53.1

^a^Scissors and locally available materials

^b^Water, butter

^c^After 5 days, 7 days and did not remember

#### Determinants of newborn care practices among postnatal mothers

In the multivariable analysis, women earn 651–1400 Ethiopian birr monthly were 57.2% less likely to practice newborn care than women who earn more than 1400 (AOR = 0.428; 95% CI 0.183, 0.999). Women who delivered spontaneously vaginally for their recent delivery were 56.2% less likely practicing newborn care for their baby as compared to women who had instrumental delivery (AOR = 0.438; 95% CI 0.240, 0.800). Regarding the history of antenatal care; these mothers who had antenatal follow up once were 88.9% less likely providing newborn care services as compared to these mothers who had four and more antenatal follow up (AOR = 0.111; 95% CI 0.013, 0.944) (Table 3).

**Table 3:** Bivariable and multivariable logistic analysis showed factors associated with poor newborn care practice among postnatal mothers at Dessie Referral Hospital, Northeast Ethiopia, 2018

Factors	Category	Newborn care practice		COR (95% CI)	AOR (95% CI)	p-value
		Good	Poor			
Age in years	15–20	6	7	10.00 (1.195, 83.691)	4.228 (0.414, 43.185)	0.224
	21–35	175	186	0.886 (0.473, 1.660)	0.861 (0.417, 1.778)	0.686
	36–49	20	24	1	1	
Educational status	Unable to read and write	22	35	1.875 (0.900, 3.904)	1.868 (0.828, 4.213)	0.132
	Able to read and write	21	16	0.898 (0.394, 2.044)	0.681 (0.267, 1.736)	0.421
	Primary education	70	102	1.717 (0.954, 3.093)	1.643 (0.873, 3.091)	0.123
	Secondary education	50	41	0.966 (0.504, 1.854)	0.995 (0.497, 0.992)	0.988
	College and above	33	28	1	1	
Monthly income	151–650 ETB	5	6	1.001 (0.300, 3.337)	0.672 (0.158, 2.868)	0.592
	651–1400 ETB	20	11	0.459 (0.214, 0.984)	0.428 (0.183, 0.999)	0.050*
	> 1401 ETB	171	205	1	1	
First ANC visit	Before 4 months	150	154	1	1	
	Four and above months	42	61	1.415 (0.900, 2.225)	0.981 (0.579, 1.663)	0.944
Knew about thermal care	Yes	146	147	1	1	
	No	50	75	1.490 (0.974, 2.278)	1.225 (0.763, 1.967)	0.400
Knew about the initiation of breastfeeding	Yes	192	209	1	1	
	No	4	13	2.986 (0.957, 9.314)	1.437 (0.352, 5.864)	0.613
Mode of delivery	SVD	130	109	0.419 (0.243, 0.722)	0.438 (0.240, 0.800)	0.007*
	Cesarean section	41	63	0.768 (0.413, 1.429)	0.912 (0.467, 1.784)	0.789
	Instrumental	25	50	1	1	
Number of ANC visit	One	143	136	0.106 (0.013, 0.845)	0.111 (0.013, 0.944)	0.044*
	Two	35	42	0.133 (0.016, 1.104)	0.151 (0.017, 1.314)	0.087
	Three	13	28	0.239 (0.027, 2.092)	0.245 (0.026, 2.298)	0.218
	Four and above	3	7	1	1	

*AOR* adjusted odd ratio, *CI* confidence interval, *COR* crude odd ratio, *ETB* Ethiopian Birr, *EBF* exclusive breastfeeding, *SVD* spontaneous vaginal delivery, *ANC* antenatal care

* Significant at p-value < 0.05

## Discussion

Our finding revealed that 46.9% (95% CI 41.9, 51.9) of postnatal mothers’ newborn care practices was good. This finding is higher than a study conducted in Mandura and Damot pulasa Woreda; the newborn care practice among postnatal mothers was 40.6% and 24% respectively [9, 13]. This might be due to the difference in study setting; facility based includes those who have good health-seeking behavior and knowledge about newborn care which increases newborn care practices. This finding is higher than a study conducted in Awobel district; revealed that the level of newborn care practices among postnatal mothers was 23.1% [15]. The possible explanation might be the socio-economic difference and Dessie is more urbanized as compared to other districts.

Our finding shows that prevalence of initiation of breastfeeding within an hour, baby bathing after 24 h, cord cutting and thermal care was 83.9%, 75.8%, 46.9%, and 80.8% respectively. This finding is similar with a study conducted in Rural Pondicherry, India; 70.6% of postnatal mothers’ bath their baby after 1 day and 84.8% of mothers gave thermal care [14] but, higher than a study conducted in Kenya [16]. Moreover, initiation of breastfeeding within an hour is higher than a study conducted in Awobel (41.6%) and Damot Pulasa Woreda (45.8%) [13, 15]. The prevalence of safe cord cutting in our study is much lower than a study finding in Awobel district (97.6%) [15].

In this study; monthly income, number of ANC visit and mode of delivery were factors associated with newborn care practices among postnatal mothers. Mothers who earn a monthly income of 651–1400 Ethiopian birr were 57.2% less likely to practice newborn care than women who earn more than 1400 Ethiopian birr and women who delivered spontaneously vaginally for their recent delivery were 56.2% less likely practicing newborn care as compared to women who had instrumental delivery. This is similar to a study conducted in eastern Uganda shows mothers in the upper quintile provide more practices to their newborns than their counterparts [17].

Moreover, mothers who had a history of antenatal follow up once were 88.9% less likely providing newborn care services as compared to those mothers who had four and more. This finding was consistent with a study done in Mandura district, Uganda, Tanzania, and Jimma [9, 17–19]. This might be due to the possibility of getting information about the components and importance of newborn care practice from healthcare providers during ANC.

### Conclusion and recommendation

This study indicated that the majority of newborn care practices are sub-optimal dominantly for home born babies. Monthly income of the respondents, antenatal care follow-up and mode of delivery were determinants for newborn care practices.

Policy makers and healthcare providers shall consider the provision of newborn care practices to prevent neonatal death especially within the first 24 h and do more on rising practices of newborn care majorly for those home born babies.

## Limitations

The major limitation of this study was facility based which affect the magnitude of newborn care practice among postnatal mothers in the community. Another limitation of this study was respondents’ bias that aims to respond to all the recommended newborn cares.

## Additional file

**Additional file 1: Table S1.** Antenatal care and delivery history of postnatal mothers at Dessie Referral Hospital, Northeast Ethiopia, 2018.

## Abbreviations

ANC: antenatal care
AOR: adjusted odds ratio
CI: confidence interval
COR: crude odds ratio
NMR: neonatal mortality rate
PNC: postnatal care
SPSS: statistical package for social science
SVD: spontaneous vaginal delivery
TT: tetanus toxoid
WHO: World Health Organization
