# Supplementary material for: Newborn care practices and its determinants among postnatal mothers in Dessie Referral Hospital, Northeast Ethiopia
Source: BMC Res Notes. 2019 Feb 21;12:96. doi: 10.1186/s13104-019-4133-3 (PMC6385447; doi:10.1186/s13104-019-4133-3)
Supplement: Supplementary file 1 — Additional file 1: Table S1. Antenatal care and delivery history of postnatal mothers at Dessie Referral Hospital, Northeast Ethiopia, 2018. [file 13104_2019_4133_MOESM1_ESM.doc]

Table S1: Antenatal care and delivery history of postnatal mothers at Dessie Referral Hospital, Northeast Ethiopia, 2018.

| Category | Category | Frequency | Percent (%) |
| --- | --- | --- | --- |
| ANC care follow up | Yes | 407 | 98.3 |
|  | No | 11 | 1.7 |
| Parity (n=418) | Primipara | 182 | 43.5 |
|  | Multiparous | 236 | 56.5 |
| When did you start ANC follow up (n=407) | Before 4 months | 295 | 72.4 |
|  | 4-7 months | 100 | 24.6 |
|  | After 7 months | 12 | 3.0 |
| How many ANC visit did you get (n=407) | Once | 279 | 68.5 |
|  | Twice | 77 | 19.0 |
|  | Three times | 41 | 10.0 |
|  | Four and above | 10 | 2.5 |
| Did you receive tetanus injections during this (or your previous) pregnancy? | Yes | 398 | 95.2 |
|  | No | 20 | 4.8 |
| Received information during ANC follow up about newborn care (n=407) | Yes | 218 | 53.6 |
|  | No | 189 | 46.4 |
| What information do you get (n=218) | Breastfeeding | 146 | 66.7 |
|  | Cord care | 9 | 4.3 |
|  | Eye care | 5 | 2.3 |
|  | Thermoregulation | 8 | 3.7 |
|  | Immunization | 50 | 23.0 |
| Who provided those information | Doctor | 38 | 17.4 |
|  | Nurse | 165 | 75.7 |
|  | Family | 7 | 3.2 |
|  | Media (e.g. new, brochures, magazines) | 5 | 2.3 |
|  | Traditional birth attendant | 3 | 1.4 |
| Planned delivery place (n=418) | Yes | 351 | 84.0 |
|  | No | 67 | 16.0 |
| Delivery place(n=418) | Health facility | 411 | 98.3 |
|  | Home | 7 | 1.7 |
| Who assisted or attended to you during delivery(n=418) | Skilled birth attendant | 415 | 99.3 |
|  | Traditional birth attendant | 3 | 0.7 |
| What was the mode of delivery? (n=418) | Spontaneous vaginal delivery | 239 | 57.2 |
|  | Cesarean section | 104 | 24.9 |
|  | Instrumental | 75 | 17.9 |
| Infants sex | Male | 213 | 51.0 |
|  | Female | 205 | 49.0 |
| what was the infants’ weight at birth or within 7 days after birth? (n=418) | Less than 2.5 kg | 88 | 21.0 |
|  | 2.5-4 kg | 299 | 71.5 |
|  | Greater than 4 kg | 14 | 3.4 |
|  | I do not know | 17 | 4.1 |
| Infants age in weeks’ (at the time of data collection) (n=418) | One | 322 | 77.0 |
|  | Two | 84 | 20.1 |
|  | Three and above | 12 | 2.9 |
